# P04.01. Complementary and alternative medicine use and health outcomes among urban adolescents with asthma

**DOI:** 10.1186/1472-6882-12-S1-P271

**Published:** 2012-06-12

**Authors:** C Luberto, S Cotton, M Yi, J Tsevat

**Affiliations:** 1 University of Cincinnati College of Medicine, Cincinnati, USA

## Purpose

Many adolescents with asthma use complementary and alternative medicine (CAM) for asthma symptom management. The purpose of this study is to examine the relationship between CAM use and health outcomes among urban adolescents with asthma.

## Methods

We examined cross-sectional and longitudinal relationships between self-reported CAM use and health outcomes in urban adolescents with asthma. Participants (Time 1: N=151; Time 2: N=132) completed questionnaires regarding the use of 10 CAM modalities following two clinic visits one year apart as part of a larger study. CAM use was dichotomized (high/low) due to its non-normal distribution. T-tests examined between-group differences in outcomes at both time points. Multivariable regression analyses using backwards elimination examined relationships between CAM use at Time 1 and health outcomes at Time 1 and Time 2, when controlling for key covariates and, in longitudinal analyses, Time 1 functioning.

## Results

Participants (mean age= 15.8 years) were 85% African-American and 60% female. Cross-sectional results demonstrated bivariate between-group differences for several health outcomes (t-score: -1.48-2.48, p<.10 to p<.01). In multivariable analyses, more frequent use of guided imagery was associated with fewer depressive symptoms (β=-.16, p<.05), and more frequent use of prayer was associated with more frequent asthma symptoms (β=-.13, p=.06). Longitudinal results demonstrated bivariate between-group differences for several health outcomes (t-score: -1.60- 3.62, p<.10 to p<.001). In multivariable analyses, more dietary changes (e.g., eating more fruit) were associated with more frequent asthma symptoms (β=-.19; p<.01). No other CAM modalities were significantly associated with health outcomes.

## Conclusion

Small but significant differences were shown for several health outcomes between high and low CAM users, though few remained significant in multivariable and/or longitudinal analyses. Most relationships involved high CAM use and poorer outcomes, though the reason for this remains unclear. Further research (e.g., randomized controlled trials) is needed to determine the safety and efficacy of CAM use for this population.

